# Comparative Analysis of Accuracy, Readability, Sentiment, and Actionability: Artificial Intelligence Chatbots (ChatGPT and Google Gemini) versus Traditional Patient Information Leaflets for Local Anesthesia in Eye Surgery

**DOI:** 10.22599/bioj.377

**Published:** 2024-08-19

**Authors:** Prakash Gondode, Sakshi Duggal, Neha Garg, Pooja Lohakare, Jubin Jakhar, Swati Bharti, Shraddha Dewangan

**Affiliations:** 1 Department of Anaesthesiology, Pain medicine and Critical Care, All India Institute of Medical Sciences (AIIMS), New Delhi, India; 2 Department of Microbiology, Mahatma Gandhi Institute of Medical Sciences (MGIMS), Wardha, India; 3 Department of Anaesthesiology, Pain medicine and Critical Care, University college of Medical Sciences (UCMS), Delhi, India

**Keywords:** AI Artificial Intelligence, Cataract, Local anesthetic, Patient education handout, Readability

## Abstract

**Background and Aim:**

Eye surgeries often evoke strong negative emotions in patients, including fear and anxiety. Patient education material plays a crucial role in informing and empowering individuals. Traditional sources of medical information may not effectively address individual patient concerns or cater to varying levels of understanding. This study aims to conduct a comparative analysis of the accuracy, completeness, readability, tone, and understandability of patient education material generated by AI chatbots versus traditional Patient Information Leaflets (PILs), focusing on local anesthesia in eye surgery.

**Methods:**

Expert reviewers evaluated responses generated by AI chatbots (ChatGPT and Google Gemini) and a traditional PIL (Royal College of Anaesthetists’ PIL) based on accuracy, completeness, readability, sentiment, and understandability. Statistical analyses, including ANOVA and Tukey HSD tests, were conducted to compare the performance of the sources.

**Results:**

Readability analysis showed variations in complexity among the sources, with AI chatbots offering simplified language and PILs maintaining better overall readability and accessibility. Sentiment analysis revealed differences in emotional tone, with Google Gemini exhibiting the most positive sentiment. AI chatbots demonstrated superior understandability and actionability, while PILs excelled in completeness. Overall, ChatGPT showed slightly higher accuracy (scores expressed as mean ± standard deviation) (4.71 ± 0.5 vs 4.61 ± 0.62) and completeness (4.55 ± 0.58 vs 4.47 ± 0.58) compared to Google Gemini, but PILs performed best (4.84 ± 0.37 vs 4.88 ± 0.33) in terms of both accuracy and completeness (p-value for completeness <0.05).

**Conclusion:**

AI chatbots show promise as innovative tools for patient education, complementing traditional PILs. By leveraging the strengths of both AI-driven technologies and human expertise, healthcare providers can enhance patient education and empower individuals to make informed decisions about their health and medical care.

## Introduction

Eye Surgeries are among the most commonly performed surgical procedures globally (Rossi et al., 2021), often eliciting strong negative emotions in patients, including fear and anxiety. This anxiety primarily stems from apprehensions related to the surgery and anaesthesia itself, fear of experiencing pain, and concerns about potential vision loss. The highest levels of negative emotions typically occur on the day of the eye surgery. To mitigate patients’ negative experiences, various strategies like preoperative education and counselling are employed. Implementing such interventions may result in improved surgical outcomes and heightened satisfaction and quality of life for patients (Obuchowska & Konopinska, 2021).

Patient education material plays a crucial role in informing and empowering individuals, particularly in specialized medical contexts such as eye surgery involving local anesthesia (WHO, 2013). Traditional sources of medical information, including online platforms like Google or Wikipedia, often present information in a manner that can overwhelm patients, leading to confusion. Moreover, conventional patient information leaflets (PILs) may offer static, one-size-fits-all information that fails to address individual patient concerns or cater to varying levels of understanding (Gal & Prigat, 2005; Coulter & Ellins, 2007).

Artificial Intelligence (AI) refers to the simulation of human intelligence in machines, enabling them to perform tasks that typically require human intelligence, such as learning, problem-solving, decision-making, and understanding natural language. AI has made significant strides across various sectors, such as healthcare and education. The emergence of AI chatbots, particularly those utilising large language models (LLMs), has unlocked novel opportunities for enriching medical training methodologies, revolutionizing the approach to preparing future healthcare professionals. These LLMs are meticulously trained on vast textual datasets sourced from the internet, specifically tailored to facilitate the creation of text-based content. AI chatbots are increasingly finding utility in healthcare contexts, such as delivering education and assistance to individuals managing chronic ailments, as well as bolstering confidence and acceptance of COVID-19 vaccines (Baglivo et al., 2023). The AI language chatbots such as Conversational/Chat Generative Pre-trained Transformer (ChatGPT) and Google Bard, now Google Gemini, present a promising avenue by providing dynamic and interactive responses to patient inquiries. Leveraging sophisticated natural language processing techniques, these chatbots can distil complex medical concepts into accessible, layman-friendly language, thereby enhancing comprehension and engagement. Unlike conventional sources, AI chatbots can tailor their responses to the unique preferences and requirements of individual patients, fostering a more personalized and user-centric approach to patient education (Pagoto & Nebeker, 2019).

This study aims to conduct a comparative analysis of the accuracy, completeness, readability, tone, and understandability of patient education material generated by AI chatbots versus traditional PILs. Specifically, the study evaluates the efficacy of AI-driven methodologies in disseminating information pertaining to local anesthesia in eye surgery, with the overarching objective of ascertaining whether AI chatbots offer a more efficient and user-friendly mechanism for conveying medical information. By harnessing the capabilities of AI technology, we aim to bridge the communication gap between medical professionals and patients, facilitating clearer communication and fostering improved healthcare outcomes.

## Methodology

Expert panel: Three professional faculties were chosen to serve as expert reviewers for evaluating the traditional and AI-generated patient education material. One faculty member specialized in ophthalmology (>15 years of experience), while the other two had extensive experience in anaesthesiology (>12 years), particularly involved in day-to-day use of local anesthesia and actively involved in patient education and counselling of patients routinely posted for eye surgeries. All selected reviewers possessed in-depth expertise and practical knowledge relevant to the subject matter, ensuring comprehensive and insightful evaluations.

Text Generation: Google Gemini and ChatGPT were tasked with generating responses to 17 frequently asked questions (FAQs) sourced from PIL designed and endorsed by the Royal College of Anaesthetists (RCoA), Association of Anaesthetists and the British Ophthalmic Anaesthesia Society (BOAS) Patient Information Leaflet (abbreviated by authors as ‘RAB’ PIL) on local anesthesia in eye surgery, available in public domain, as a traditional patient information leaflet available freely on the internet (RCOA, 2023).

Blinding Annotation: To ensure impartial evaluation, the generated responses were blinded as Annexure A (ChatGPT), Annexure B (Gemini), and Annexure C (RAB). The order of presentation for each annexure was randomized to mitigate order effects.

Accuracy and Completeness Rating: The expert reviewers independently rated the accuracy and completeness of each FAQ response on a 5-point Likert scale, based on the extent to which the information aligns with established medical knowledge and covers all relevant aspects of the topic. The scores of each were collected to find out the mean scores and were later used for further analysis.

Readability Assessment: Readability was assessed using established scores, including the Flesch-Kincaid Grade Level (FKGL), Gunning Fog Index (GFI), and Flesch Reading Ease (FRE) scores. These measures ensure that the text is accessible to a wide range of readers (Rouhi et al., 2024). These readability metrics are primarily based on factors like the average number of words per sentence, the number of complex words, and the average number of syllables per word.

Sentiment Analysis: Sentiment analysis was conducted to determine the emotional tone of each response (positive, neutral, or negative), providing insights into the overall sentiment conveyed by the patient education material generated by each AI chatbot (Kiritchenko, Zhu & Mohammad, 2014).

PEMAT (Patient Education Materials Assessment Tool) Score Calculation: The PEMAT score serves as a crucial instrument in evaluating the quality and accessibility of patient education materials. Created by the Agency for Healthcare Research and Quality (AHRQ), PEMAT assesses both the understandability and actionability of these materials, ensuring they effectively convey information to patients and empower them to take appropriate actions regarding their health (AHRQ, 2020). By utilizing the PEMAT score, healthcare providers and educators can enhance the clarity and effectiveness of patient education materials, fostering improved patient comprehension and engagement with healthcare information. The score can range from 1% to 100%. A combined score of 70% or above for both understandability and actionability is typically considered indicative of high-quality patient education material (Shoemaker, Wolf & Brach, 2014).

Ethics Declaration: Ethical clearance was not sought for this study as it involves evaluating patient education material generated by publicly available AI language chatbots and does not entail direct interaction with human subjects or access to sensitive personal information. The study focuses on assessing the quality and efficacy of patient education material rather than conducting research involving human subjects, and all data analysed are anonymized, ensuring participant confidentiality and the absence of personally identifiable information. Nonetheless, the research adheres to ethical principles by prioritizing participant confidentiality, voluntary participation, and ensuring no harm to human subjects.

Statistical analysis: The data was collected and recorded in Microsoft® Excel® 2021 MSO (Version 2403 Build 16.0.17425.20124), and results were evaluated using Stata® software version 15.0, and online statistical tests calculator (see appendix). The data’s normality was checked using the Kolmogorov-Smirnov test of normality. Normally distributed variables (accuracy-completeness scores, readability scores, and the PEMAT scores) were described as mean (±SD) and compared using the Analysis of Variance (ANOVA) test. A *P* value of <0.05 was considered significant. Tukey Honestly Significant Difference (HSD) was used for post hoc analysis. Microsoft® Excel® was used to generate graphical representations of various data sets.

AI use Disclosure: During the preparation of this work, the authors used AI chatbots ChatGPT and Google Gemini in order to generate the response to the FAQs for further analysis, comparing them to the traditional PIL. After using this tool, the authors reviewed and edited the content as needed and takes full responsibility for the content of the publication.

Permissions: Appropriate permissions were sought and granted from the Royal College of Anaesthetists (RCoA) for the utilization of their patient information leaflets in this study. We express our heartfelt appreciation to the RCoA for generously providing access to their invaluable patient information leaflets. These leaflets have greatly enriched our study by offering comprehensive insights into local anaesthesia education in eye surgery. We are sincerely grateful for the RCoA ‘s commitment to patient care and education, as well as their willingness to share their resources for the benefit of research and healthcare advancement.

## Results

Readability Assessment: The readability of the text generated by AI language chatbots (ChatGPT and Google Gemini) and the Royal College of Anaesthetists’ Patient Information Leaflet (RAB PIL) was evaluated using established readability metrics. The Flesch-Kincaid Grade Level (FKGL) aims to assign a specific grade level based on the complexity of the text. It indicates the readability level based on the US grade school system. Lower FKGL scores suggest easier readability. For instance, an FKGL score of 0–3 would indicate kindergarten/elementary, and a basic reading level would correspond to the age group of 5–8 years. Similarly, an FKGL score of 9–12 means an average reading level corresponding to a high school age range of 14–17 years. While a score of 12–15 would indicate advanced reading levels corresponding to college levels, and an age group of 17–20 years. The Gunning Fog Index (GFI) primarily focuses on the complexity of sentences rather than individual words. The GFI formula generates a grade level between 0 and 20. A GFI score of 6 is easily readable for sixth-graders. A score above 17 means a graduate level. Text for general public should aim for a grade level of around 8. The Flesch Reading Ease (FRE) score typically ranges between 1 and 100, with higher scores indicating easier readability and lower scores suggesting more difficult reading, unlike the FKGL and GFI where higher scores indicate difficult readability. Usually, a readability score of 60 and above is standard reading levels easily understood by 13- to 15-year-old students. The results are summarized in the table (Table 1).

**Table 1:** Readability scores.


READABILITY SCORES	ChatGPT	GEMINI	RAB

Flesch-Kincaid Grade Level	10	8.5	8.6

Gunning Fog Index	13.9	11.4	12.1

Flesch Reading Ease	47.5	51.3	57.8

Reach	85%	96%	96%

Tone	formal	formal	formal

Personalism	personal	personal	personal


ChatGPT: Conversational Generative Pre-trained Transformer. Gemini: Google Gemini AI Chatbot. RAB: Royal College of Anaesthetists patient information leaflet.

FKGL: Google Gemini demonstrated the lowest FKGL score of 8.5, followed closely by RAB PIL and ChatGPT with scores of 8.6 and 10, respectively. GFI: Google Gemini exhibited the lowest GFI score of 11.4, suggesting slightly simpler language compared to both ChatGPT and RAB PIL. FRE: RAB PIL demonstrated the highest FRE score of 57.8, followed by Google Gemini with a score of 51.3, and ChatGPT with a score of 47.5. The readability reach indicates the percentage of the general population likely to comprehend the text. Both Google Gemini and RAB PIL achieved a readability reach of 96%, indicating broad accessibility. ChatGPT’s text reached 85% of the general population, slightly lower than the other two sources. Overall, all sources maintained a formal tone and a personal approach, enhancing engagement and understanding among readers.

Sentiment Analysis: The sentiment analysis of the text generated by ChatGPT, Google Gemini, and RAB PIL revealed varying emotional tones. The sentiment scores range from –100 to +100, where –10 to +10 indicates a neutral tone, while a more negative score indicates a serious tone, and a more positive score indicates an enthusiastic tone (Table 2). Google Gemini exhibited the highest sentiment score of +73.7, indicating a predominantly positive emotional tone characterized by enthusiasm. The text generated by Google Gemini conveyed optimism and positivity, fostering a favourable reader perception. On the other hand, ChatGPT demonstrated the lowest sentiment score of –53.2, suggesting a predominantly negative emotional tone. Despite the serious tone, the sentiment conveyed by ChatGPT’s text was notably more negative, potentially affecting reader engagement and receptivity. RAB PIL, although maintaining a serious tone similar to ChatGPT, presented a sentiment score of –23.2, indicating a somewhat negative emotional tone. While maintaining a formal and serious approach, the text’s sentiment leaned slightly towards the negative spectrum, potentially influencing reader perception. Overall, Google Gemini’s text exhibited the most positive sentiment, while ChatGPT’s text had a more negative sentiment, and RAB PIL’s sentiment fell somewhere in between, highlighting the importance of emotional tone in patient education materials.

**Table 2:** Sentiment/Emotional analysis.


	ChatGPT	GEMINI	RAB

Sentiment score	–53.2	+73.7	–23.2

Effect tone	Serious	Enthusiastic	Serious

Sentiment Tone	Quite negative	Quite positive	Somewhat negative


ChatGPT: Conversational Generative Pre-trained Transformer. Gemini: Google Gemini Ai Chatbot. RAB: Royal College of Anaesthetists patient information leaflet.

PEMAT Scores for Understandability and Actionability: The Patient Education Materials Assessment Tool (PEMAT) scores for understandability and actionability of the FAQs related to local anesthesia in eye surgery are summarized in Table 3. ANOVA results revealed significant differences in understandability scores across the three sources (*p* = 0.005), as well as actionability scores (*p* = 0.012), indicating variations in the effectiveness of conveying information among ChatGPT, Google Gemini, and RAB PIL. The post hoc analysis revealed significant differences in understandability between ChatGPT and RAB PIL (*p* = 0.006) but not between Google Gemini and RAB PIL (*p* = 0.237). Post hoc results for actionability revealed significant differences between both comparisons of ChatGPT versus RAB (0.015) and Gemini versus RAB (0.049). These findings suggest that while Google Gemini may perform comparably to RAB PIL on understanding, ChatGPT demonstrates superior understandability and actionability in patient education materials related to local anesthesia in eye surgery.

**Table 3:** Patient Education Materials Assessment Tool (PEMAT) Scores for Understandability and Actionability.


PEMAT SCORES		ChatGPT		GEMINI		RAB	
		
FAQs		U	A	U	A	U	A

1.	What a local anaesthetic is?	90	85	85	85	80	80

2.	What are the advantages of local anaesthesia?	95	95	90	90	90	90

3.	Who will give the anaesthetic?	85	90	85	85	85	85

4.	How is the local anaesthetic given?	90	85	90	85	80	80

5.	Can everyone have a local anaesthetic for an eye operation?	85	85	85	85	85	85

6.	I would prefer a general anaesthetic; do I have a choice?	90	90	90	90	85	85

7.	Are there any complications from local anaesthetics?	85	85	90	90	85	85

8.	Will the local anaesthetic injection hurt?	85	85	90	90	85	85

9.	How will I know that the anaesthetic is working?	90	90	90	90	85	85

10.	Will I feel anything during the operation?	90	90	85	85	90	90

11.	Will I see anything during the operation?	90	90	90	90	80	80

12.	What will I hear during the operation?	90	90	90	90	85	85

13.	Can I wear my hearing aid during the operation?	85	85	85	85	85	85

14.	What if I want to wriggle, cough, clear my throat or scratch my nose?	85	85	85	85	85	85

15.	When can I go home?	95	95	95	95	90	90

16.	Will I be in pain?	90	90	85	85	85	85

17.	Do I need to take any special care?	95	95	95	95	90	90

	Mean ± SD	89.12 ± 3.6	88.82 ± 3.8	88.53 ± 3.4	88.24 ± 3.5	85.29 ± 3.3	85.29 ± 3.3

P value for Understandability 0.005		P value for Actionability – 0.012					

**POST HOC TUKEY HSD ANALYSIS**							

Understandability		Actionability					

ChatGPT vs RAB (p-value = 0.006)		ChatGPT vs RAB (p-value = 0.015)					

Gemini vs RAB (p-value = 0.236)		Gemini vs RAB (p-value = 0.049)					


ChatGPT: Conversational Generative Pre-trained Transformer. Gemini: Google Gemini AI Chatbot. RAB: Royal College of Anaesthetists patient information leaflet. Score range: 1–100. (U: understandability score, A: actionability score) Significant if *p* < 0.05.

**Figure 1:**
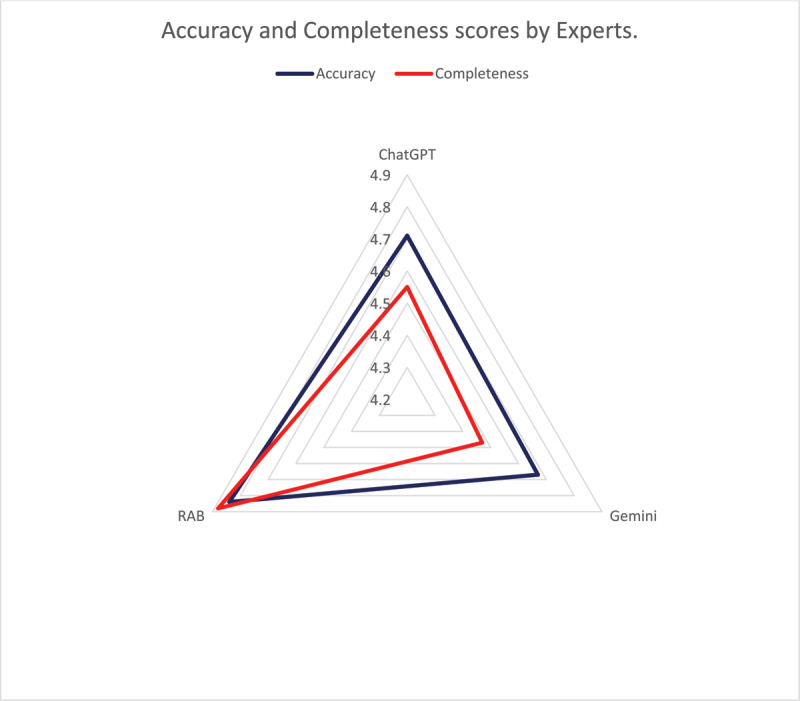
Radar Graph for Accuracy and Completeness. ChatGPT: Conversational Generative Pre-trained Transformer. Gemini: Google Gemini AI Chatbot. RAB: Royal College of Anaesthetists patient information leaflet. Both Accuracy and Completeness are higher for RAB PIL.

Expert Analysis for Accuracy and Completeness: The accuracy and completeness of the responses provided by ChatGPT, Google Gemini, and the Royal College of Anaesthetists’ Patient Information Leaflet (RAB PIL) were meticulously evaluated by three experts. Each FAQ was individually rated on a Likert scale ranging from 1 to 5, with higher scores indicating higher levels of accuracy and completeness. The mean scores after blinded evaluation are given in the table (Table 4). ANOVA results revealed no significant differences in accuracy scores (*p* = 0.186), but significant differences in completeness scores (*p* < 0.001) across the three sources. The post hoc analysis revealed a significant difference in completeness between ChatGPT and RAB PIL (*p* = 0.003), and similarly between Gemini and RAB PIL (*p* = 0.0002), but not between ChatGPT and Google Gemini (*p* = 0.716). No significant differences were observed in accuracy scores between any pair of sources. These findings suggest that while ChatGPT may offer slightly higher accuracy and completeness compared to Gemini, RAB PIL performs best in terms of both accuracy and completeness in providing patient education material on local anesthesia in eye surgery (Figure 1).

**Table 4:** Experts rating for accuracy and completeness expressed as Mean ± Standard deviation.


	ChatGPT	GEMINI	RAB	P-VALUE

Accuracy	4.71 + –0.5	4.67 + –0.6	4.84 + –0.4	0.186

Completeness	4.55 + –0.6	4.47 + –0.6	4.88 + –0.3	<0.001

Post Hoc Tukey HSD Analysis				

ChatGPT VS GEMINI (p value = 0.716)				

ChatGPT VS RAB PIL(p value = 0.003)				

GEMINI VS RAB PIL (p value < 0.001)				


ChatGPT: Conversational Generative Pre-trained Transformer. Gemini: Google Gemini AI Chatbot. RAB: Royal College of Anaesthetists patient information leaflet. Score expressed as Mean ± SD. Significant if *p* < 0.05.

## Discussion

The results of this study highlight several key findings. Firstly, the readability analysis revealed variations in the complexity and ease of comprehension among the texts generated by different sources. While AI chatbots may offer simplified language, traditional PILs still maintain an edge in terms of overall readability and accessibility. Our findings are similar to a study that compared traditional patient education materials with ChatGPT for men’s health (Shah et al., 2024).

Sentiment analysis further revealed differences in the emotional tone conveyed by the patient education material generated by AI chatbots and traditional PILs. Google Gemini exhibited a predominantly positive sentiment, characterized by enthusiasm, whereas ChatGPT’s text conveyed a more negative sentiment. RAB PIL fell somewhere in between, leaning slightly towards a negative emotional tone. These findings underscore the importance of emotional tone in patient education material, as it can significantly impact reader engagement and receptivity. The study by Machova *et al*. (2023) underscores the potential of machine learning in emotion detection but acknowledges the ongoing challenge of fully automating this process.

The Patient Education Materials Assessment Tool (PEMAT) scores provided insights into the understandability and actionability of the patient education material. While no significant differences were found in accuracy scores between the three sources, variations were observed in completeness scores. RAB PIL consistently outperformed AI chatbots in terms of completeness, suggesting that traditional PILs may provide more comprehensive information. However, AI chatbots demonstrated superior understandability and actionability, indicating their potential to convey information in a clear and actionable manner. A recent study by Cheong *et al*. (2024) evaluating patient education materials for obstructive sleep apnoea (OSA) found that ChatGPT provided more comprehensive information than Google Gemini (formerly Google Bard).

This study offers valuable insights into the varying performance of AI-driven patient education material compared to traditional PILs and highlights the factors contributing to these differences. The disparities in scores among the texts of ChatGPT, Google Gemini, and RAB PIL, can be attributed to several key factors. Firstly, the nature of content generation plays a significant role. AI chatbots like ChatGPT and Google Gemini rely on natural language processing algorithms to generate responses, whereas traditional PILs like RAB PIL are meticulously crafted through a collaborative effort involving anaesthesiologists, eye surgeons, patients, and patient representatives. This collaborative process ensures that PILs are thoroughly vetted, comprehensive, and reflective of established medical knowledge and patient preferences (Elwyn et al., 2006). In contrast, AI-generated content may lack the depth, and contextual understanding that human experts provide, leading to variations in accuracy, completeness, and tone (Holzinger et al., 2019). Secondly, the inherent differences in the underlying technologies contribute to variations in performance. AI chatbots leverage machine learning algorithms trained on vast datasets to generate responses, allowing for scalability, adaptability, and real-time interaction. However, these algorithms may struggle with complex medical concepts or nuanced language, leading to inaccuracies or misinterpretations (Shortliffe & Sepulveda, 2018). Traditional PILs, while static and less interactive, benefit from human expertise and validation, ensuring reliability and accuracy (Houts et al., 2006).

Additionally, the study’s focus on a specific medical context, namely local anesthesia in eye surgery, may influence the performance of AI chatbots and traditional PILs. Complex medical procedures require precise, detailed, and contextually relevant information to ensure patient understanding and safety. While AI chatbots offer the advantage of dynamic, personalized responses tailored to individual queries, they may struggle to provide the level of detail and accuracy required for complex medical topics. In contrast, traditional PILs are designed to provide comprehensive information in a structured format, making them well-suited for conveying complex medical concepts. Also, variables like age, gender, literacy, area of residence (Urban/Rural), technological comprehension, and information literacy may impact the use of AI chatbots.

Moreover, the accessibility and ease of use of AI chatbots compared to complex medical websites play a significant role in their selection as patient education tools. Navigating medical websites for accurate and reliable information can be daunting and time-consuming for patients, especially those with limited health literacy or digital skills (Kontos et al., 2014). AI chatbots offer a user-friendly interface, real-time interaction, and simplified language, making them more accessible and engaging for patients seeking medical information.

**Figure 2:**
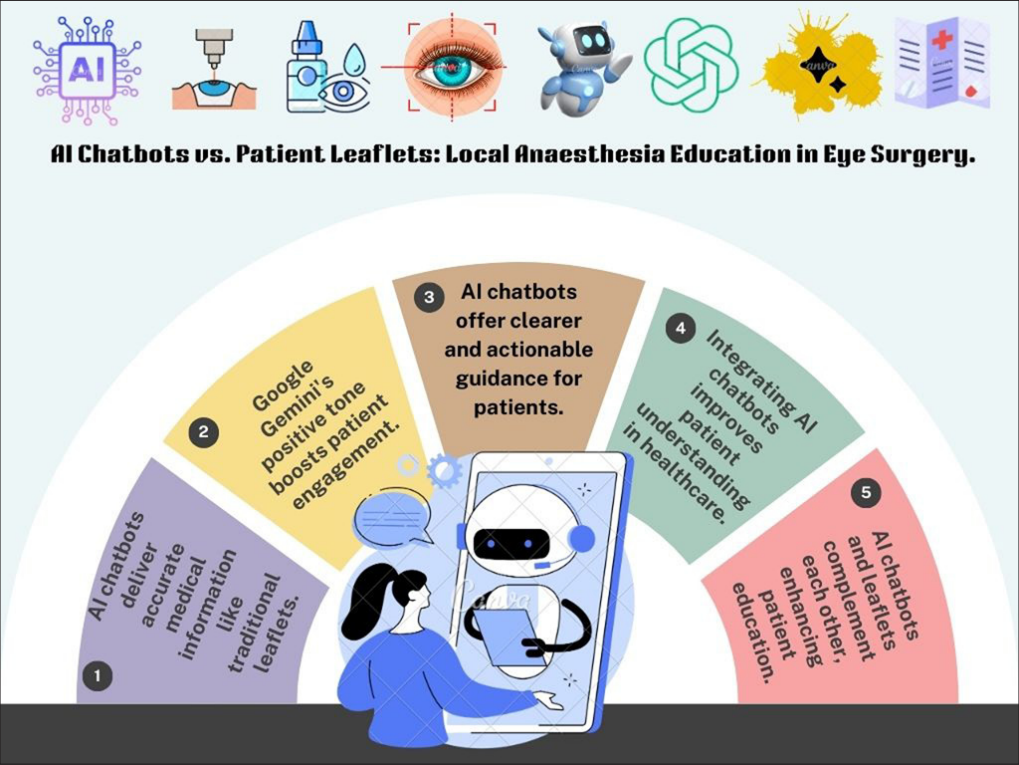
Illustration of positive findings and strength of the study. AI: Artificial Intelligence. Chatbots: ChatGPT (Conversational Generative Pre-trained Transformer) and Gemini (Google Gemini). Traditional leaflet: Royal College of Anaesthetists patient information leaflet.

Strengths: The study showcases innovation in healthcare communication by employing AI chatbots for patient education. It offers a thorough analysis comparing AI-generated material with traditional PILs, providing valuable insights. Methodologically robust, it integrates expert evaluation, readability assessment, sentiment analysis, and PEMAT scores for comprehensive evaluation. Ethical considerations are diligently addressed, ensuring participant confidentiality and adherence to ethical research practices. Relevant study strengths and positive findings are illustrated in the figure (Figure 2).

Limitations: Scope constraints may limit the generalizability of findings beyond the specific medical context studied. Standardized metrics may not fully capture patient understanding or emotions. The small number of expert reviewers raises concerns about the statistical robustness. Expert ratings are subjective and may be influenced by biases. Future research could use qualitative methods for better insights. However, the findings’ applicability beyond the specific context may be limited by cultural and healthcare differences. Caution should be exercised when extrapolating the results to other populations or medical domains. AI technology could introduce biases (Ferrara, 2023) and question result validity, especially in rapidly evolving technological landscapes. Another limitation is that we did not consider how revising the prompts might affect the quality of the refined responses.

Future Prospects: Future research could focus on optimizing AI chatbots for enhanced accuracy, completeness, readability, and emotional tone in patient education. Integrating patient feedback after proper ethical approval and real-world data could improve relevance and effectiveness. Longitudinal studies are crucial for assessing AI chatbots’ long-term impact on patient understanding, engagement, and healthcare outcomes. Integrating AI chatbots into healthcare systems could improve patient-provider communication and decision-making. Interdisciplinary collaboration is essential for developing ethically sound and clinically relevant AI-driven patient education tools for real-world application.

Conclusion: AI chatbots show promise as innovative tools for patient education, complementing traditional PILs, their optimization remains a key focus for future research. They have the potential to supplement the standard patient education materials, but currently are not a substitute for the latter. Longitudinal studies are essential to assess their long-term impact on patient understanding, engagement, and healthcare outcomes. By integrating the strengths of AI-driven technologies with human expertise, healthcare providers can bridge the communication gap between medical professionals and patients, empowering individuals to make informed decisions about their health and medical care.

## Appendix: Websites and software used

For finding the FAQs for comparison, the website of Royal College of Anaesthetists was accessed at: https://rcoa.ac.uk/sites/default/files/documents/2023-09/06-EyeOperation2023web.pdf.

For generating text, we used the chatbots of ChatGPT 3.5, freely available in public domain, available at: https://https://chat.openai.com/ and google Gemini available at: https://https://gemini.google.com/app.

For readability, we used the online calculator available at: https://readable.com/ and https://readabilityformulas.com/readability-scoring-system.php.

For calculating the sentiment score we used online sentiment tone analysis tools available at: https://monkeylearn.com/sentiment-analysis-online/ and https://www.danielsoper.com/sentimentanalysis/default.aspx.

For calculating statistics, Microsoft® Excel® 2021 MSO (Version 2403 Build 16.0.17425.20124), online software available at: https://www.socscistatistics.com/tests/anova/default2.aspx and Stata software® version15.0.
